# Biochemical and Biophysical Properties of Red Blood Cells in Disease

**DOI:** 10.3390/biom12070923

**Published:** 2022-07-01

**Authors:** Gregory Barshtein

**Affiliations:** Department of Biochemistry, The Faculty of Medicine, Campus Een Kerem, The Hebrew University of Jerusalem, Jerusalem 91120, Israel; gregoryba@ekmd.huji.ac.il

Red blood cells (RBCs, erythrocytes) are highly specialized cells devoted to the transport of respiratory gases. The nature of the membrane of erythrocytes, as well as proteins of the cytoskeleton, their molecular interactions, the lipid composition of this membrane, the content of ions and water, membrane permeability, and the regulation of signaling pathways through specific receptors, are different aspects that determine the unique properties of red blood cells.

RBCs are subjected to blood flow-induced shear stress and oxidative stress in blood circulation. RBCs have unique biochemical and biophysical properties to withstand these conditions, which define their functionality. In a mature state in a healthy person, these cells live in circulation for 100 to 120 days. However, in numerous pathological conditions (e.g., diabetes, cardiovascular disease, hemoglobinopathies, malaria) and under aging (during storage and in-vivo), RBCs’ biochemical and biophysical features can be drastically altered. These alterations can be used as a marker of pathology.

## 1. RBC as a Marker of Pathology

The idea of using this or that property of RBCs as a marker of a pathological state is not new. One of the relevant diagnostic markers, determined routinely in clinical practices, is erythrocyte sedimentation rate (ESR) [1]. The ESR has long been used as a “sickness indicator” due to its reproducibility and low cost. Normally, RBCs settle relatively slowly, with a rate of ≤20 mm/h. A faster-than-normal rate may indicate inflammation that may also signify a chronic disease, an immune disorder, or other medical condition. Rabe et al. tested whether the ESR can be used as a biomarker to monitor the treatment response of ChAc patients to dasatinib and explain the influence of particular plasma components and cellular properties on a putative change in the ESR. Authors performed mathematical modeling to dissect the contributions of RBCs shape and rigidity to a decreased ESR and demonstrated that the rigidity of the RBCs is the dominating parameter and RBCs shape plays just a minor role.

The primary aspiration of two studies presented by Werning et al. and Todinova et al. in this issue is directed toward discovering novel biomarkers for disease diagnostics and progression of neurodegenerative diseases (NDDs). The pathological features of NDDs suggest that diagnostic markers can be found in the blood. In the present issue, this idea is discussed in two papers (Werning et al. and Todinova et al.). While Werning et al. were studying the carboxylate metabolism of RBCs obtained from pantothenate kinase-associated neurodegeneration (PKAN) patients, Todinova et al. were conducting coulometric studies of red cells from NDDs patients.

PKAN is a progressive NDD caused by mutations in the pantothenate kinase 2 (PANK2) gene and associated with iron deposition in basal ganglia. Werning et al. revealed the altered metabolism of carboxylates in the red cells of patients suffering from PKAN and described the molecular effects of PANK2 point mutants on the enzyme’s expression/stability and activity. Todinova et al. investigated the thermodynamic behavior of RBCs obtained from patients with three common NDD (Parkinson’s disease, Alzheimer’s disease, and amyotrophic lateral sclerosis) and compared it with that in healthy subjects. Todinova et al. found that NDD can be distinguished from a normal healthy state based on changes in the thermodynamic parameters of the unfolding of the main proteins of RBCs (cytoplasmic hemoglobin and membrane protein band-3).

In this issue, Pavlou et al. presented the study of coagulation abnormalities in renal pathology of chronic kidney disease. Specifically, the authors demonstrated a substantial elevation of RBC aging markers (phosphatidylserine externalization and intracellular reactive oxygen species concentration) in end-stage renal disease.

Thus, we can conclude that erythrocytes are very sensitive to changes in the external environment, and monitoring RBC properties can help diagnose and evaluate the effectiveness of their treatment.

## 2. In-Vivo and In-Vitro Aging of RBC

### 2.1. In-Vivo RBC Aging

The aging of red blood cells (RBCs) in-vivo represents a key biomedical issue. Normal human RBCs all survive to about the same age, which implies the existence of a molecular countdown that triggers a series of changes leading to removal by the reticuloendothelial system. Several senescence markers are known that tag RBCs as senescent and prime them for clearance [2]. Age-related alterations occurring on the surface of the RBC membrane can lead to a change in the cell’s affinity for plasma proteins. Thus, Semenov et al. demonstrated that cells from the younger fractions (obtained by Percoll density gradient) could bind significantly more fibrinogen than older erythrocytes. The authors discussed the features of the interaction of fibrinogen macromolecules with the red cell membrane and confirmed its contribution to the mechanisms of RBC aggregation. The obtained knowledge is essential for understanding the fundamental mechanisms of fibrinogen-induced erythrocyte aggregation.

### 2.2. RBC Storage and Transfusion

Blood transfusion is a routine life-saving therapy that has revolutionized medicine [3]. Blood donations for transfusion are routinely stored as packed RBC (PRBC) for up to 35 or 42 days, depending on the preservation solution [4]. In the last decade, many studies have reported the negative outcome of PRBC transfusion in various groups of patients [5,6,7,8,9]. Some studies attributed this to the storage-induced lesion to RBC, while others did not find a correlation with the storage duration [6,10,11,12]. Senescence lesion markers appear on the transfused cells, shortening the RBC lifespan in circulation. In the specific context of sickle-cell disease, senescence signals can also trigger painful occlusive events typical of the disease (Chadebech et al.). Several studies reported impairment of RBC functionality during routine storage [13,14,15,16,17], which becomes significant at about two weeks of storage [14,16,18], with substantial unit-to-unit variability [14]. Livshits et al. suggest that part of the indicated deterioration in the PRBC’s functionality (during storage) may be associated with cell glycolysis due to high sugar concentration in the storage medium.

Following removal of the highly damaged cells [19], most of the transfused RBCs (in the range of 75% [20]) remain in the bloodstream of the recipient in an environment substantially different from the one in which they were stored [21]. As a result, the remaining transfused RBCs can partially restore their functionality and remain in the recipient’s blood for a more extended period.

It was also established that the process of concentrates preparation leads to a change in the properties of PRBC [15]. Considering this possibility, Schiroli et al. compared the properties of concentrates obtained by two alternative procedures. In method-1, blood was leucodepleted and then separated to get PRBCs, while in method-2, blood was separated and leucodepleted after removing plasma and buffy coat. Properties of PRBCs were tested after 2, 7, and 14 days of storage. The authors have established that RBCs count and Hb content were found to be higher in method-1, while PRBCs obtained with method-2 contained less K^+^, iron, and storage lesions markers.

After their storage, PRBCs are transfused to the recipient, but we do not clearly understand what happens to PRBCs after they are transfused to the recipient. The part (from several to ten percent) of highly damaged transfused RBCs is removed from the bloodstream immediately or in the first hours later [19]. The remaining RBCs are exposed to the environment (blood plasma of the recipient). This impact can lead to a partial restoration of the functionality of transfused cells [22] or can lead to their additional aging (Chadebech et al.). In this case, the direction of the effect will depend on the state of the recipient’s blood to whom the packed cells were transfused. Thus, the level of oxidative stress in the recipient’s blood can be an essential factor in the effectiveness of PRBC transfusion. Chadebech et al., using RBC biochemical (phosphatidylserine externalization, Ca^2+^ influx) and biophysical (adhesion to thrombospondin) markers, characterize the effect of sickle anemia patients’ plasma on the functionality of packed RBCs. The authors demonstrated that incubation (during 48 h) of PRBCs with sickle-cell disease patient plasma leads to an intracellular influx of Ca^2+^, externalization of phosphatidylserine, and elevation of red cells adhesion to thrombospondin (Chadebech et al.).

Thus, we can assume that a set of factors determines the behavior of transfused cells in the recipient’s bloodstream. These may include the initial properties of the donor cells, the method of concentrate preparation, the storage conditions of the cells, and, finally, the level of oxidative stress in the recipient’s plasma.

## 3. Conclusions

We hope that the presented Special Issue, which focuses on RBCs’ biophysical/biochemical properties, will contribute to the knowledge about RBCs and their role in human health. We thank all the participants in the collection who presented their exciting publications. We also hope that the topic we have raised will attract many new researchers.
